# The relationship between sleep duration and obesity risk among school students: a cross-sectional study in Zhejiang, China

**DOI:** 10.1186/s12986-018-0285-8

**Published:** 2018-07-09

**Authors:** Hao Wang, Ruying Hu, Huaidong Du, Bragg Fiona, Jieming Zhong, Min Yu

**Affiliations:** 1 Department of NCDs Control and Prevention, Zhejiang Provincial Center for Diseases Control and Prevention, #3399 Binsheng road, Binjiang District, Hangzhou, Zhejiang Province China; 2 Clinical Trial Service Unit & Epidemiological Studies Unit (CTSU), Nuffield Department of Population Health, University of Oxford, Oxford, UK; 3 Medical Research Council Population Health Research Unit, Nuffield Department of Population Health, University of Oxford, Oxford, UK

**Keywords:** Sleep duration, Overweight, Obesity, Adolescents, Factors

## Abstract

**Background:**

Obesity has been identified as a major risk factor for a large number of chronic diseases. Understanding factors related to adolescent obesity is critical for prevention of chronic diseases. The associations between sleep duration and obesity among adolescents in the existing literature are controversial. Our study was designed to determine the prevalence of short sleep duration, and assess the association of sleep duration and obesity, among middle and high school students in Zhejiang, China.

**Methods:**

18,403 Students in 442 schools were recruited and surveyed using an anonymous, self-administered questionnaires. Weighted multivariable logistic regression models were used for data analyses.

**Results:**

The mean (SD) age of the students was 15.9 (1.8) years. 49.7% of students were girls. The mean (SD) height and weight were 166.2 (8.5) cm and 54.6 (11.1) kg, respectively. The overall prevalence of obesity and overweight were 3.4% (95% CI: 3.0–3.8) and 7.8% (95% CI: 7.4–8.3), respectively. The overall prevalence of short sleep duration among students was 66.0% (95% CI: 63.8–68.1), higher among girls than boys (69.8% vs. 62.1%) (*P* < 0.0001). The figures for middle school, academic high school, and vocational high school were 59.0, 82.4 and 59.7%, respectively (*P* < 0.0001). As compared with girls who sleep 8 h per day (reference), the odds ratios (95% CI) of obesity for girls who sleep < 7 h, 7 h, 9 h and ≥ 10 h were 1.97 (1.15–3.38), 1.90 (1.18–3.04), 1.38 (0.86–2.20) and 2.12 (1.22–3.67) respectively, after adjustment for socio-demographic status, lifestyle factors, and mental health. The corresponding figures among boys were 1.45 (0.97–2.16), 1.13 (0.81–1.57), 1.25 (0.89–1.74), and 1.12 (0.81–1.54), respectively.

**Conclusions:**

Insufficient sleep is prevalent among students in Zhejiang China. A U-shaped relationship was found between sleep duration and obesity risk among girls, with the lowest risk among those who slept for 8 h, but not among boys. Adequate sleep duration may be an important component of obesity prevention initiatives among adolescents.

**Electronic supplementary material:**

The online version of this article (10.1186/s12986-018-0285-8) contains supplementary material, which is available to authorized users.

## Background

Worldwide, the prevalence of overweight and obesity have steadily increased over the last several decades, with the number of obese individuals estimated to reach 711.4 million in 2015, of which 107.7 million were children [1]. Globally, high BMI contributed to 4 million deaths in 2015, equivalent to 7.1% of all-cause deaths, and 120 million disability-adjusted life years (DALYs), accounting for 4.9% of DALYs from any cause among adults [1]. In many countries, obesity rates among children are rising faster than among adults, and this is particularly true in China, which has the highest number of obese children [2].

In China, the prevalence of overweight and obesity among 7–18-year students increased from 1.1 to 9.6% and from 0.1 to 4.9%, respectively, between 1985 and 2010, and in 2010 the prevalence of obesity varied from 0.4 to 21.7% across different provinces in China [3]. Zhejiang Province, situated in the east of China, has a population of 56 million. During the past decade, with increasing urbanization and changes in lifestyle, the prevalence of overweight and obesity among adolescents has increased dramatically, reaching 10.6 and 5.3%, respectively in 2010 [4], higher than the Chinese national average.

Overweight and obesity are well-documented risk factors for a large number of chronic diseases, including diabetes, cancers, strokes, and coronary heart disease [5–7]. Hence, understanding factors related to adolescent obesity is critical for addressing this urgent public health concern. A wide range of factors, including low physical activity, prolonged sedentary time, and unhealthy dietary behaviours, have been found to be adversely associated with adolescent obesity [8–10], and there is a growing literature documenting the relationship of sleep and obesity. However, most studies examining the association of sleep with adiposity among adolescents are from western countries [11, 12], where the patterns of adolescent sleep and obesity may be different from China [13, 14]. Furthermore, the associations between sleep duration and obesity among adolescents in the existing literature remain controversial. While some studies report null associations between sleep duration and obesity [11, 15, 16], others have found U-shaped [12, 17] or negative linear [18] relationships between sleep duration and obesity. A study of 66,817 10–18-year adolescents from China found a U-shaped association of sleep duration and obesity [19], and the current study was designed with the aim of examining the prevalence of short sleep duration and assessing the relationship of sleep duration with obesity among students in Zhejiang Province, China.

## Methods

### Sample and procedure

**Fig. 1:**
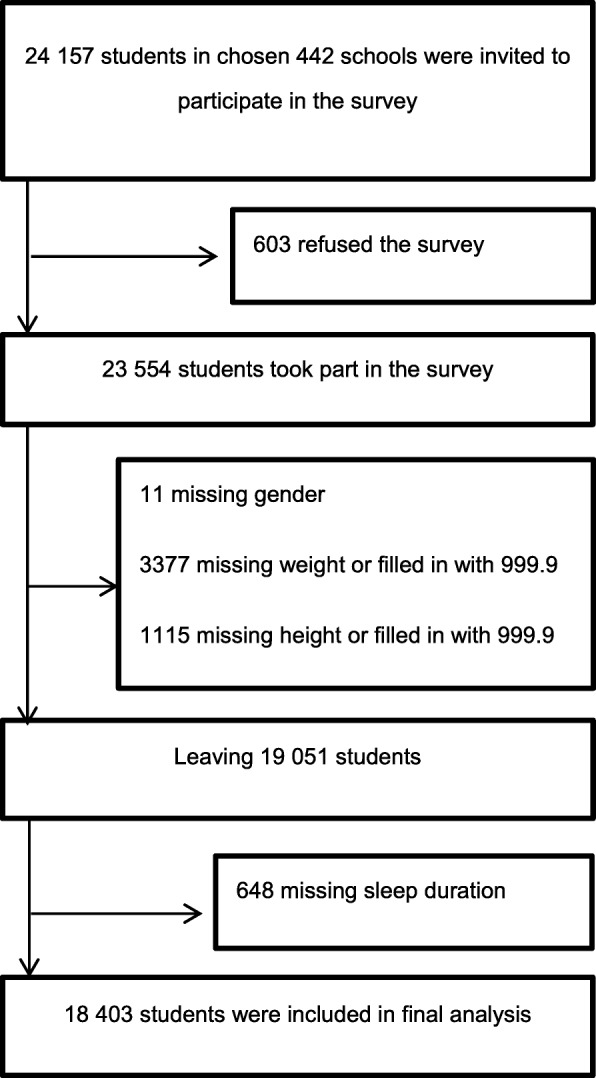
Flowchart of participants included in final analysis

Details of study design, sample, and participants have been reported previously [20]. Briefly, during April and May 2017, a total of 24,157 students from grades 7–12 in 442 different schools in 30 counties in Zhejiang were invited to participate, with 23,554 students participating in the survey (response rate: 97.5%). Written informed consent was obtained from all participants and their guardians before the survey. After exclusion of subjects with missing key variables (including sex, height, weight and sleep duration), 18,403 eligible subjects were included in the final analyses (Fig. 1), of whom, 9259 (49.7%) were girls and the mean (±SD) age was 15.9 ± 1.8 years. 8834 (47.7%) participants were middle school students, 5597 (29.0%) were academic high school students and 3972 (23.3%) came from vocational high school. The survey questionnaire was based on the Youth Risk Behaviour Survey, developed by the Centers for Disease Control and Prevention (CDCs) [21], and the international Global School-based Student Health Survey (GSHS), supported by the World Health Organization [22]. The questionnaire covered demographic characteristics (birth year and month, parental education), tobacco use, alcohol use, dietary consumption (breakfast, fruit, vegetables, milk, and carbonated beverages), physical activity, screen-time and mental health (loneliness). The self-administrated questionnaire was completed anonymously by students and put directly into sealed boxes after completion. In order to improve the response rate, every recruited student was given a pencil box as a gift. The study design and procedure was approved by the ethics committee of Zhejiang Provincial CDC.

### Measures

#### Outcome variables

Height was assessed by the question “How tall are you without your shoe on? □□□.□ cm (write your height in the blank boxes. If you do not know or refuse, please fill in the blank boxes with 999.9” Weight was assessed by the question “How much do you weigh without your shoes on? □□□.□ kg (write your weight in the blank boxes. If you do not know or refuse, please fill in the blank boxes with 999.9” Body mass index (BMI) was calculated as the ratio of self-reported weight (kg) to the square of self-reported height (m). Standard cut-offs for adolescent overweight and obesity (Table 1), established by Chinese Working Group on Obesity for Children (WGOC) [23], were used in the analyses.

**Table 1:** Standard cut-offs for overweight and obesity in Chinese children and adolescents

Age(years)	Overweight		Obesity	
	Boys	Girls	Boys	Girls
7-	17.4	17.2	19.2	18.9
8-	18.1	18.1	20.3	19.9
9-	18.9	19.0	21.4	21.0
10-	19.6	20.0	22.5	22.1
11-	20.3	21.1	23.6	23.3
12-	21.0	21.9	24.7	24.5
13-	21.9	22.6	25.7	25.6
14-	22.6	23.0	26.4	26.3
15-	23.1	23.4	26.9	26.9
16-	23.5	23.7	27.4	27.4
17-	23.8	23.8	27.8	27.7
18-	24.0	24.0	28.0	28.0

#### Exposure variable

Sleep duration was assessed through the question: “During the past 30 days, on average, how long did you sleep every day (including daytime rest)? □□ Hours □□ Minutes” Sleep duration (hours) was calculated as: hours + (minutes/60). Short sleep duration was defined as sleep duration < 9 h per day for children aged 6–12 years or < 8 h per day for teens aged ≥13 years, as recommended by American Academy of Sleep Medicine (AASM) [24].

#### Other covariates

Some covariate question and response options (including parental education, cigarette use, alcohol use, physical activity, screen-time, and loneliness) have been reported previously [20]. Breakfast was assessed by the question: “During the past 7 days, on how many days did you eat breakfast?” Answer options included “None”, “1 day”, “2 days”, “3 days”, “4 days”, “5 days”, “6 days”, and “7 days”. Answers were further categorized into two groups: “Daily” and “Nondaily”. Fruit intake was assessed by the question: “During the past 30 days, how many times per day did you usually eat fruit, such as apples, oranges, mangoes, or papayas?” Response options included “None”, “< 1 time”, “1 time”, “2 times”, “3 times”, “4 times”, and “≥ 5 times”. Answers were further categorized into two groups: “Daily” and “Nondaily”. Vegetable intake was assessed by the question: “During the past 30 days, how many times per day did you usually eat vegetables, such as cauliflower, cabbage?” Response options for vegetable intake were identical to those for fruit. Answers were further categorized into two groups: “≥2 times/day” and “< 2 times/day”. Milk intake was assessed by the question: “During the past 30 days, on how many days per week did you drink milk?” Response options included “None”, “< 1 day”, “1–2 days”, “3–4 days” and “5–7 days”. Options were further divided into two groups: “< 3 days/week” and “≥3 days/week”. Carbonated beverages were assessed by the question: “During the past 30 days, how many times per day did you usually drink carbonated soft drinks, such as Coca-Cola, Pepsi, or Sprite? (Do not include diet soft drinks.)”. Response options included “None”, “1–3 times/week”, “4–6 times/week”, “1 time/d”, “2 times/d”, “3 times/d”, and “≥ 4 times/d”. Options were further divided into two groups: “< 4 times/week” and “≥4 times/week”.

### Statistical analysis

All analyses were performed using SAS software V.9.3. A weighting factor was applied to each student record to adjust for non-response and for the varying probabilities of selection. The weight used for estimation in this survey is given by: W = W1 × W2 × f1 × f2. W1 = the inverse of the probability of selecting the county. W2 = the inverse of the probability of selecting the classroom within the county. f1 = a student-level nonresponse adjustment factor calculated by class. f2 = a post-stratification adjustment factor calculated by grade [25]. Continuous variables were given as mean ± standard deviation. The prevalence of short sleep duration and overweight/obesity were given as percentage and 95% confidence intervals (CI). Weighted prevalence and weighted mean were calculated using the PROC SURVEYFREQ, and PROC SURVEYMEAN procedure, respectively. Comparison of weighted prevalence was performed using Rao-Scott x^2^, and comparison of weighted mean was performed using design based linear regression.

To examine the associations of sleep duration and obesity, weighted multivariable logistic regression analyses were performed using the PROC SURVEYLOGISTC procedure. In multivariable logistic regression, sleep duration (continuous) was transformed into a categorical variable comprising five groups: “< 7 h”, “7 h”, “8 h”, “9 h”, and “≥10 h”. Students who slept 8 h per day were selected as reference group. Three logistic regression models were fitted to assess adjusted odds ratios (ORs) and their 95% CI. In model 1, ORs were adjusted for age group (≤13 y, 14–15 y, and ≥ 16 y), sex (boys and girls), area (urban and rural), types of school (middle school, academic high school, and vocational high school), paternal and maternal education level (high school or below, college or above, and unknown). In model 2, ORs were additionally adjusted for cigarette use (current smoker and non-smoker), alcohol use (current drinker and non-drinker), breakfast consumption (daily and nondaily), fruit consumption (daily and nondaily), vegetable consumption (≥2 times/day and < 2 times/day), milk consumption (≥3 days/week and < 3 days/week), carbonated drinks consumption (≥4 times/week and < 4 times/week), physical activity (daily and nondaily), and screen-time (≥2 h/day and < 2 h/day). In model 3, ORs were additionally adjusted for loneliness (never/occasionally, sometimes and often/always). All statistical tests were two tailed, and *P*-values < 0.05 were considered statistically significant.

## Results

### Descriptive statistics

The percentage of students whose fathers and mothers were educated to college level or above were 12.2 and 10.7% (Table 2), respectively. Compared to students with non-short sleep duration, students with short sleep duration had lower physical activity (19.4% vs.15.1%) (*P* < 0.0001), shorter screen-time (24.2% vs.16.6%) (*P* < 0.0001), less frequent consumption of breakfast (74.0% vs.69.4%) (*P* < 0.0001) and fruits (74.7% vs. 69.8%) (*P* < 0.0001), and more frequent feelings of loneliness (7.4% vs. 13.2%) (*P* < 0.0001). Mean student height and weight were 166.2 ± 8.5 cm and 54.6 ± 11.1 kg, respectively. Mean sleep duration was 8.3 ± 1.6 h per day. Comparison of included and excluded participants showed no statistically significant differences for the majority of variables examined (Additional file 1: Table S1), but compared to included students, those excluded from the study were more likely to be younger, to be boys, to attend middle school, and to use electronic screen devices, and were less likely to have well-educated parents and to drink alcohol.

**Table 2:** Characteristics of adolescents from Zhejiang by sleep duration

Characteristics	Total	Non-short	Short	*P* value
		sleep duration	sleep duration	
	(*N* = 18,403)	(*N* = 6184)	(*N* = 12,219)	
Age (years)	15.9 ± 1.8	15.8 ± 1.8	15.9 ± 1.8	0.33
Girls (%)	9259 (49.7)	2774 (44.1)	6485 (52.7)	<.0001
Urban (%)	7314 (33.1)	2133 (29.6)	5181 (34.9)	0.05
Types of school (%)				<.0001
Middle school	8834 (47.7)	3651 (57.4)	5183 (42.6)	
Academic high school	5597 (29.0)	922 (15.0)	4675 (36.3)	
Vocational high school	3972 (23.3)	1611 (27.6)	2361 (21.1)	
Paternal education level (%)				<.0001
High school or below	10,525 (81.5)	5046 (82.3)	9636 (81.0)	
College or above	6787 (12.2)	684 (9.7)	1946 (13.6)	
Unknown	1091 (6.3)	454 (8.0)	637 (5.4)	
Maternal education level (%)				<.0001
High school or below	11,352 (82.7)	5054 (82.4)	9875 (82.9)	
College or above	5880 (10.7)	619 (9.0)	1684 (11.6)	
Unknown	1171 (6.6)	511 (8.6)	660 (5.5)	
Physically active daily (%)	3090 (16.6)	1230 (19.4)	1860 (15.1)	<.0001
Screen duration ≥2 h per day (%)	3462 (19.2)	1521 (24.2)	1941 (16.6)	<.0001
Consuming breakfast daily (%)	13,036 (70.9)	4587 (74.0)	8449 (69.4)	<.0001
Consuming fruits daily (%)	13,170 (71.5)	4648 (74.7)	8522 (69.8)	<.0001
Consuming vegetables ≥2 times daily (%)	13,746 (74.7)	4559 (73.6)	9187 (75.3)	0.08
Consuming milk ≥3 days weekly (%)	12,211 (66.9)	4164 (67.9)	8047 (66.3)	0.07
Consuming carbonated drinks ≥4 times weekly (%)	2389 (12.7)	779 (12.2)	1610 (12.9)	0.35
Current cigarette smoking (%)	992 (5.5)	371 (5.9)	621 (5.3)	0.29
Current alcohol drinking (%)	4311 (23.6)	1400 (23.1)	2911 (23.8)	0.57
Often/always feel lonely (%)	2055 (11.2)	447 (7.4)	1608 (13.2)	<.0001
Height (cm)	166.2 ± 8.5	165.6 ± 8.8	166.5 ± 8.3	<.0001
Weight (kg)	54.6 ± 11.1	53.7 ± 11.5	55.0 ± 10.8	<.0001
BMI (kg/m^2^)	19.7 ± 3.3	19.5 ± 3.5	19.8 ± 3.1	<.0001
Sleep duration (hours)	8.3 ± 1.6	9.7 ± 1.0	7.6 ± 0.8	<.0001

Mean and number in brackets were weighted

*BMI* body mass index

### Prevalence of obesity and overweight

The overall prevalence of obesity and overweight were 3.4% (95% CI: 3.0–3.8) and 7.8% (95% CI: 7.4–8.3), respectively (Table 3). The prevalence of obesity among boys was higher than among girls (4.3% vs. 2.4%) (*P* < 0.0001). The corresponding figures for overweight were 10.4 and 5.3%, respectively (*P* < 0.0001). The prevalence of overweight in urban areas was higher than in rural areas (9.3% vs. 7.1%). The prevalence of obesity among middle school, academic high school, and vocational high school students were 4.1, 2.2, and 3.6%, respectively, with corresponding figures for overweight of 7.4, 8.2, and 8.3%, respectively.

**Table 3:** Comparison of weighted prevalence of overweight and obesity between different groups

Characteristics	Overweight/obesity			Overweight			Obesity		
	Prevalence (%)^a^	x^2b^	*P* value	Prevalence (%)^a^	x^2b^	*P* value	Prevalence (%)^a^	x^2b^	*P* value
Sex		189.6	<.0001		104.3	<.0001		41.1	<.0001
Boys	14.7 (13.7–15.7)			10.4 (9.7–11.1)			4.3 (3.8–4.9)		
Girls	7.7 (7.1–8.3)			5.3 (4.6–5.9)			2.4 (2.1–2.8)		
Area		11.0	0.0009		25.6	<.0001		0.0001	0.99
Urban	12.7 (11.6–13.9)			9.3 (8.5–10.1)			3.4 (2.7–4.1)		
Rural	10.5 (9.7–11.3)			7.1 (6.6–7.6)			3.4 (2.9–3.9)		
Types of school		3.2	0.2		3.2	0.2		25.2	<.0001
Middle school	11.4 (10.5–12.3)			7.4 (6.8–8.0)			4.1 (3.5–4.7)		
Academic high school	10.4 (9.2–11.5)			8.2 (7.3–9.1)			2.2 (1.7–2.6)		
Vocational high school	12.0 (10.4–13.5)			8.3 (7.2–9.5)			3.6 (2.9–4.3)		

^a^Based on the weighted data. ^b^ Rao-Scott x^2^

### Prevalence of short sleep duration

The overall prevalence of short sleep duration was 66.0% (95% CI: 63.8–68.1), significantly higher among girls than boys (69.8% vs. 62.1%) (*P* < 0.0001), and marginally higher in urban, than rural, areas (69.5% vs. 64.2%) (*P* = 0.05) (Table 4). The prevalence of short sleep duration among middle school, academic high school, and vocational high school students were 59.0, 82.4 and 59.7%, respectively (*P* < .0001). The prevalence of short sleep duration increased with the increasing age (*P* < .0001).

**Table 4:** Comparison of weighted prevalence of short sleep duration by different characteristics

Characteristics	Prevalence (%)^a^	x^2^	*P* value
Age (y)		885.8^b^	<.0001
≤ 13	44.1 (40.9–47.3)		
14–15	68.5 (65.7–71.3)		
≥ 16	72.5 (70.1–74.9)		
Sex		43.4^c^	<.0001
Boys	62.1 (59.9–64.4)		
Girls	69.8 (67.2–72.4)		
Areas		3.77^c^	0.05
Urban	69.5 (66.0–73.1)		
Rural	64.2 (61.0–67.4)		
Types of school		158.5^c^	<.0001
Middle school	59.0 (55.8–62.2)		
Academic high school	82.4 (79.7–85.1)		
Vocational high school	59.7 (56.4–63.0)		

^a^Based on the weighted data. ^b^ Trend x^2^. ^c^ Rao-Scott x^2^

### Associations of sleep duration and obesity

After adjustment for socio-demographic status (Table 5), compared to girls who slept 8 h per day, those who slept < 7 h, 7 h, 9 h, and ≥ 10 h per day had 86% (OR = 1.86, 95% CI: 1.08–3.22), 82% (OR = 1.82, 95% CI: 1.14–2.92), 40% (OR = 1.40, 95% CI: 0.87–2.24), and 109% (OR = 2.09, 95% CI: 1.23–3.58), respectively higher risk for obesity in model 1. After additional adjustment for lifestyle factors, girls who slept < 7 h, 7 h, 9 h, and ≥ 10 h per day had 96% (OR = 1.96, 95% CI: 1.14–3.35), 89% (OR = 1.89, 95% CI: 1.18–3.03), 38% (OR = 1.38, 95% CI: 0.87–2.19), and 113% (OR = 2.13, 95% CI: 1.23–3.69), respectively greater odds for obesity risk compared with the reference group. After further adjustment for loneliness, the corresponding odds ratios (95% CI) were 1.97 (1.15–3.38), 1.90 (1.18–3.04), 1.38 (0.86–2.20), and 2.12 (1.22–3.67), respectively. A U-shaped relationship was observed between sleep duration and obesity among adolescent girls. However, no statistically significant difference was found among adolescent boys. The corresponding odds ratios (95% CI) for boys were 1.45 (0.97–2.16), 1.13 (0.81–1.57), 1.25 (0.89–1.74) and 1.12 (0.81–1.54), respectively.

**Table 5:** Adjusted odds ratios for obesity according to sleep duration among students in Zhejiang, China

	< 7 h	7 h	8 h	9 h	≥10 h
Overall					
Total	2025	4262	6279	3225	2612
Obese	74	119	184	124	119
Model 1	1.52 (1.08–2.12) *	1.30 (0.99–1.71)	1.00	1.31 (0.99–1.73)	1.40 (1.05–1.89) *
Model 2	1.55 (1.10–2.19) *	1.34 (1.02–1.76) *	1.00	1.30 (0.98–1.71)	1.39 (1.03–1.88) *
Model 3	1.60 (1.14–2.23) ^&^	1.36 (1.03–1.78) *	1.00	1.29 (0.97–1.70)	1.39 (1.02–1.87) *
Female					
Total	1109	2394	3176	1485	1095
Obese	29	50	58	44	43
Model 1	1.86 (1.08–3.22)	1.82 (1.14–2.92)	1.00	1.40 (0.87–2.24)	2.09 (1.23–3.58)
Model 2	1.96 (1.14–3.35) *	1.89 (1.18–3.03) ^&^	1.00	1.38 (0.87–2.19)	2.13 (1.23–3.69) ^&^
Model 3	1.97 (1.15–3.38) *	1.90 (1.18–3.04) ^&^	1.00	1.38 (0.86–2.20)	2.12 (1.22–3.67) ^&^
Male					
Total	916	1868	3103	1740	1517
Obese	45	69	126	80	76
Model 1	1.37 (0.92–2.03)	1.07 (0.77–1.50)	1.00	1.27 (0.91–1.76)	1.13 (0.82–1.56)
Model 2	1.40 (0.93–2.10)	1.11 (0.80–1.54)	1.00	1.25 (0.90–1.74)	1.12 (0.81–1.54)
Model 3	1.45 (0.97–2.16)	1.13 (0.81–1.57)	1.00	1.25 (0.89–1.74)	1.12 (0.81–1.54)

Model 1, adjusted for age group, sex, areas, types of school, paternal and maternal education level. Model 2, additionally adjusted for cigarette use, alcohol use, breakfast consumption, fruit consumption, vegetable consumption, milk consumption, carbonated drinks consumption, physical activity and screen-time. Model 3, additionally adjusted for loneliness

*P < 0.05; ^&^*P* < 0.01

## Discussion

This provincially representative survey shows that two-thirds of students experienced insufficient sleep duration. The association between sleep duration and obesity differed by sex; after controlling for socio-demographic status, lifestyle factors, and mental health, a U-shaped relationship was found between sleep duration and obesity among girls, but not among boys.

### Overweight/obesity

Our study found that the prevalence of overweight/obesity derived from self-reported height and weight was 11.2%, lower than in another study of 33,256 7–18-year students conducted in Zhejiang in 2010, which showed a prevalence, derived from objectively-measured data, of 15.9% [4]. Previous studies have documented that self-reported weight and height might underestimate BMI [26–28]. A study of 24,221 students from 8th and 11th grade in the United States compared the difference between self-reported and objectively-measured height and weight, and found students overestimated their height by 0.68–2.02 cm, and underestimated their weight by 0.4–0.98 kg [29]. However, when direct measurement of height and weight is not practical, self-reported measurements are suggested to provide a reliable proxy measure among adolescents [29]. In addition, previous studies showed that self-reported height and weight information collected from girls tends to result in greater underestimation of BMI than self-reported height and weight information collected from boys [30, 31], which may explain why the prevalence of overweight and obesity among girls was lower than among boys in our study (overweight: 5.3% vs. 10.4%; obesity: 2.4% vs. 4.3%).

### Short sleep duration

Globally, increased prevalence of insufficient sleep seems to parallel increased prevalence of overweight and obesity [32]. Our study showed that, although lower than that reported in the United States in 2015 (72.7%) [33], the overall prevalence of short sleep duration among middle and high school students was 66.0% in Zhejiang, suggesting two-thirds of students were facing consequences of sleep deficiency. One possible reason for this high prevalence is competition pressure from entrance exams, in particular the college entrance exam which is usually viewed as the most important and highly competitive exam in China. This is supported by our observation that compared with students from middle school (59.0%) and vocational high school (59.7%), students from academic high school had the highest prevalence of short sleep duration (82.4%).

### Relationship of obesity and short and long sleep duration

More recently, a meta-analysis of prospective studies including 26,652 adolescents aged 12–18 years from 3 different studies in the US, found that short sleep duration was a risk factor or marker (RR = 1.3, 95% CI: 1.11–1.53) for the development of obesity among adolescents [34], consistent with our findings that sleep deficiency was positively related to obesity risk. The explanation for the association between short sleep duration and obesity remains unclear, but there are several possible mechanisms explaining the association. First, short sleep duration influences the secretion of a number of hormones, and leads to decreased leptin [35] and increased ghrelin [36] levels, both of which are associated with increased appetite [37, 38]. In addition, it is hypothesised that tiredness caused by short sleep duration might lead to lower levels of physical activity, in turn promoting weight gain [39, 40]. This hypothesis was validated by our finding that students with short sleep duration were less physically active than those with non-short sleep duration (15.1% vs. 19.4%).

However, contrary to another cross-sectional study showing short sleep duration was associated with obesity only in adolescent boys [41], the positive association in our study was found only among girls, and not among boys. This discrepancy may reflect smaller sample size, less comprehensive adjustment for covariates relating to obesity, ethnic differences, sleep duration category differences, and failure to consider mental health in the analyses in this study. A European study of 3311 adolescents aged 12–17 years indicated that adolescents who slept for shorter duration had higher BMI, body fat and waist circumference, particularly among females [42], consistent with our study. A study of 66,817 adolescents aged 10 to 18 years from China found that the adjusted ORs (95% CI) of obesity for students reporting < 5.0 h, 5.0–6.9 h and ≥ 9.0 h of sleep were 1.24 (0.97–1.57), 0.94 (0.87–1.01), and 1.42 (1.24–1.63), respectively, in comparison with their counterparts who slept 7.0–8.9 h daily, and the study’s authors recommended an optimal sleep duration of 7.0–8.0 h daily for prevention of adolescent overweight/obesity [19]. By contrast, our study showed that girls who slept 7 h per day had a 90% higher risk of obesity comparing with those who slept 8 h daily, suggesting that sleeping 7.0–7.9 h daily might also have adverse effect on adolescent health, and that 8.0 h was the minimum time that middle and high school students need to sleep every day to experience a lower risk of obesity. This finding has very important public health implications, and provides evidence to inform guidance on sleep duration among adolescents.

Meanwhile, our study found that students with a sleep duration ≥10 h per day had 39% greater odds of obesity compared with those with sleep duration of 8 h per day. The possible mechanisms underlying the association of prolonged sleep duration with obesity among adolescents remain unclear. One possible explanation is that prolonged sleep duration reflects compensation for poor-quality and/or interrupted sleep, which may be more common among overweight and obese person. A study showed that, compared with normal-weight children, overweight and obese children had less slow-wave sleep (SWS), which plays a major role in body restorative processes and energy metabolism [43].

In the present study, a U-shaped association was found between sleep duration and risk of obesity only among girls, and not among boys. This sex-difference may reflect sex-differences in the physiology of puberty, particularly in body composition. For example, during puberty, boys rapidly increase muscle mass and reduce body fat mass due to increase of testosterone and growth hormone, while girls increase fat mass due to increase of estradiol [44].

Our study had several strengths. First, it included a large and provincially representative sample. Second, a wide range of covariates including socio-demographic status, lifestyle factors (e.g., cigarettes, alcohol, dietary habit, physical activity and screen-time) and mental health (loneliness) were adjusted for in analyses examining the associations of sleep duration and obesity. However, our study also had several limitations. First, the cross-sectional design limits causal inference. Second, all data was self-reported by participants and not objectively measured, which might increase the risk of information bias, including through underestimation of BMI. Third, although analyses were adjusted for most established potential confounding factors, residual confounding might still exist.

## Conclusion

In summary, our study found a U-shaped relationship of sleep duration and obesity among girls, but not among boys, and 8 h sleep was associated with the lowest risk of obesity among middle and high school students.

## Additional file

Additional file 1: **Table S1.** Comparison of characteristics between included and excluded participants. (DOCX 32 kb)

## Abbreviation

AASM: American academy of sleep medicine
BMI: body mass index
CDCs: centers for disease control and prevention
CI: confidence interval
DALYs: disability-adjusted life years
GSHS: global school-based student health survey
ORs: odds ratios
SD: standard deviation
WGOC: working group on obesity for children
