# Supplementary material for: The relationship between sleep duration and obesity risk among school students: a cross-sectional study in Zhejiang, China
Source: Nutr Metab (Lond). 2018 Jul 9;15:48. doi: 10.1186/s12986-018-0285-8 (PMC6038205; doi:10.1186/s12986-018-0285-8)
Supplement: Supplementary file 1 — Table S1. Comparison of characteristics between included and excluded participants. (DOCX 32 kb) [file 12986_2018_285_MOESM1_ESM.docx]

**Table 1. Standard cut-offs for overweight and obesity in Chinese children and adolescents**

| Age(years) | Overweight | |  | Obesity | |
| --- | --- | --- | --- | --- | --- |
|  | Boys | Girls |  | Boys | Girls |
| 7- | 17.4 | 17.2 |  | 19.2 | 18.9 |
| 8- | 18.1 | 18.1 |  | 20.3 | 19.9 |
| 9- | 18.9 | 19.0 |  | 21.4 | 21.0 |
| 10- | 19.6 | 20.0 |  | 22.5 | 22.1 |
| 11- | 20.3 | 21.1 |  | 23.6 | 23.3 |
| 12- | 21.0 | 21.9 |  | 24.7 | 24.5 |
| 13- | 21.9 | 22.6 |  | 25.7 | 25.6 |
| 14- | 22.6 | 23.0 |  | 26.4 | 26.3 |
| 15- | 23.1 | 23.4 |  | 26.9 | 26.9 |
| 16- | 23.5 | 23.7 |  | 27.4 | 27.4 |
| 17- | 23.8 | 23.8 |  | 27.8 | 27.7 |
| 18- | 24.0 | 24.0 |  | 28.0 | 28.0 |

**Table 2. Characteristics of adolescents from Zhejiang by sleep duration**

| Characteristics | Total | Non-short | Short | *P* value |
| --- | --- | --- | --- | --- |
|  |  | sleep duration | sleep duration |  |
|  | (N=18403) | (N= 6184) | (N= 12219) |  |
| Age (years) | 15.9±1.8 | 15.8±1.8 | 15.9±1.8 | 0.33 |
| Girls (%) | 9259 (49.7) | 2774 (44.1) | 6485 (52.7) | <.0001 |
| Urban (%) | 7314 (33.1) | 2133 (29.6) | 5181 (34.9) | 0.05 |
| Types of school (%) |  |  |  | <.0001 |
| Middle school | 8834 (47.7) | 3651 (57.4) | 5183 (42.6) |  |
| Academic high school | 5597 (29.0) | 922 (15.0) | 4675 (36.3) |  |
| Vocational high school | 3972 (23.3) | 1611 (27.6) | 2361 (21.1) |  |
| Paternal education level (%) |  |  |  | <.0001 |
| High school or below | 10 525 (81.5) | 5046 (82.3) | 9636 (81.0) |  |
| College or above | 6787 (12.2) | 684 (9.7) | 1946 (13.6) |  |
| Unknown | 1091 (6.3) | 454 (8.0) | 637 (5.4) |  |
| Maternal education level (%) |  |  |  | <.0001 |
| High school or below | 11 352 (82.7) | 5054 (82.4) | 9875 (82.9) |  |
| College or above | 5880 (10.7) | 619 (9.0) | 1684 (11.6) |  |
| Unknown | 1171 (6.6) | 511 (8.6) | 660 (5.5) |  |
| Physically active daily (%) | 3090 (16.6) | 1230 (19.4) | 1860 (15.1) | <.0001 |
| Screen duration ≥2 hours per day (%) | 3462 (19.2) | 1521 (24.2) | 1941 (16.6) | <.0001 |
| Consuming breakfast daily (%) | 13 036 (70.9) | 4587 (74.0) | 8449 (69.4) | <.0001 |
| Consuming fruits daily (%) | 13 170 (71.5) | 4648 (74.7) | 8522 (69.8) | <.0001 |
| Consuming vegetables ≥2 times daily (%) | 13 746 (74.7) | 4559 (73.6) | 9187 (75.3) | 0.08 |
| Consuming milk ≥3 days weekly (%) | 12 211 (66.9) | 4164 (67.9) | 8047 (66.3) | 0.07 |
| Consuming carbonated drinks ≥4 times weekly (%) | 2389 (12.7) | 779 (12.2) | 1610 (12.9) | 0.35 |
| Current cigarette smoking (%) | 992 (5.5) | 371 (5.9) | 621 (5.3) | 0.29 |
| Current alcohol drinking (%) | 4311 (23.6) | 1400 (23.1) | 2911 (23.8) | 0.57 |
| Often/always feel lonely (%) | 2055 (11.2) | 447 (7.4) | 1608 (13.2) | <.0001 |
| Height (cm) | 166.2±8.5 | 165.6±8.8 | 166.5±8.3 | <.0001 |
| Weight (kg) | 54.6±11.1 | 53.7±11.5 | 55.0±10.8 | <.0001 |
| BMI (kg/m^2^) | 19.7±3.3 | 19.5±3.5 | 19.8±3.1 | <.0001 |
| Sleep duration (hours) | 8.3±1.6 | 9.7±1.0 | 7.6±0.8 | <.0001 |

Mean and number in brackets were weighted

BMI: body mass index.

**Table 3. Comparison of weighted prevalence of overweight and obesity between different groups**

| Characteristics | Overweight/obesity | | | |  | | Overweight | | | |  | Obesity | | |
| --- | --- | --- | --- | --- | --- | --- | --- | --- | --- | --- | --- | --- | --- | --- |
|  | Prevalence (%)* | x^2^# | *P* value | |  | | Prevalence (%)* | | x^2^# | *P* value |  | Prevalence (%)* | x^2^# | *P* value |
| Sex |  | 189.6 | <.0001 | | |  | |  | 104.3 | <.0001 |  |  | 41.1 | <.0001 |
| Boys | 14.7 (13.7-15.7) |  |  |  | 10.4 (9.7-11.1) | | | |  |  |  | 4.3 (3.8-4.9) |  |  |
| Girls | 7.7 (7.1-8.3) |  |  |  | 5.3 (4.6-5.9) | | | |  |  |  | 2.4 (2.1-2.8) |  |  |
| Area |  | 11.0 | 0.0009 |  |  | | | | 25.6 | <.0001 |  |  | 0.0001 | 0.99 |
| Urban | 12.7 (11.6-13.9) |  |  |  | 9.3 (8.5-10.1) | | | |  |  |  | 3.4 (2.7-4.1) |  |  |
| Rural | 10.5 (9.7-11.3) |  |  |  | 7.1 (6.6-7.6) | | | |  |  |  | 3.4 (2.9-3.9) |  |  |
| Types of school |  | 3.2 | 0.2 |  |  | | | | 3.2 | 0.2 |  |  | 25.2 | <.0001 |
| Middle school | 11.4 (10.5-12.3) |  |  |  | 7.4 (6.8-8.0) | | | |  |  |  | 4.1 (3.5-4.7) |  |  |
| Academic high school | 10.4 (9.2-11.5) |  |  |  | 8.2 (7.3-9.1) | | | |  |  |  | 2.2 (1.7-2.6) |  |  |
| Vocational high school | 12.0 (10.4-13.5) |  |  |  | 8.3 (7.2-9.5) | | | |  |  |  | 3.6 (2.9-4.3) |  |  |

*Based on the weighted data. #: Rao-Scott x^2^.

**Table 4. Comparison of weighted prevalence of short sleep duration by different characteristics**

| Characteristics | Prevalence (%)* | x^2^ | *P* value |
| --- | --- | --- | --- |
| Age (y) |  | 885.8$ | <.0001 |
| ≤13 | 44.1 (40.9-47.3) |  |  |
| 14-15 | 68.5 (65.7-71.3) |  |  |
| ≥16 | 72.5 (70.1-74.9) |  |  |
| Sex |  | 43.4# | <.0001 |
| Boys | 62.1 (59.9-64.4) |  |  |
| Girls | 69.8 (67.2-72.4) |  |  |
| Areas |  | 3.77# | 0.05 |
| Urban | 69.5 (66.0-73.1) |  |  |
| Rural | 64.2 (61.0-67.4) |  |  |
| Types of school |  | 158.5# | <.0001 |
| Middle school | 59.0 (55.8-62.2) |  |  |
| Academic high school | 82.4 (79.7-85.1) |  |  |
| Vocational high school | 59.7 (56.4-63.0) |  |  |

*Based on the weighted data. $: Trend x^2^. #: Rao-Scott x^2^.

**Table 5. Adjusted odds ratios for obesity according to sleep duration among students in Zhejiang, China**

|  | <7 h | 7h | 8h | 9h | ≥10 h |
| --- | --- | --- | --- | --- | --- |
| Overall |  |  |  |  |  |
| Total | 2025 | 4262 | 6279 | 3225 | 2612 |
| Obese | 74 | 119 | 184 | 124 | 119 |
| Model 1 | 1.52 (1.08-2.12) * | 1.30 (0.99-1.71) | 1.00 | 1.31 (0.99-1.73) | 1.40 (1.05-1.89) * |
| Model 2 | 1.55 (1.10-2.19) * | 1.34 (1.02-1.76) * | 1.00 | 1.30 (0.98-1.71) | 1.39 (1.03-1.88) * |
| Model 3 | 1.60 (1.14-2.23) & | 1.36 (1.03-1.78) * | 1.00 | 1.29 (0.97-1.70) | 1.39 (1.02-1.87) * |
| Female |  |  |  |  |  |
| Total | 1109 | 2394 | 3176 | 1485 | 1095 |
| Obese | 29 | 50 | 58 | 44 | 43 |
| Model 1 | 1.86 (1.08-3.22) | 1.82 (1.14-2.92) | 1.00 | 1.40 (0.87-2.24) | 2.09 (1.23-3.58) |
| Model 2 | 1.96 (1.14-3.35) * | 1.89 (1.18-3.03) & | 1.00 | 1.38 (0.87-2.19) | 2.13 (1.23-3.69) & |
| Model 3 | 1.97 (1.15-3.38) * | 1.90 (1.18-3.04) & | 1.00 | 1.38 (0.86-2.20) | 2.12 (1.22-3.67) & |
| Male |  |  |  |  |  |
| Total | 916 | 1868 | 3103 | 1740 | 1517 |
| Obese | 45 | 69 | 126 | 80 | 76 |
| Model 1 | 1.37 (0.92-2.03) | 1.07 (0.77-1.50) | 1.00 | 1.27 (0.91-1.76) | 1.13 (0.82-1.56) |
| Model 2 | 1.40 (0.93-2.10) | 1.11 (0.80-1.54) | 1.00 | 1.25 (0.90-1.74) | 1.12 (0.81-1.54) |
| Model 3 | 1.45 (0.97-2.16) | 1.13 (0.81-1.57) | 1.00 | 1.25 (0.89-1.74) | 1.12 (0.81-1.54) |

Model 1, adjusted for age group, sex, areas, types of school, paternal and maternal education level. Model 2, additionally adjusted for cigarette use, alcohol use, breakfast consumption, fruit consumption, vegetable consumption, milk consumption, carbonated drinks consumption, physical activity and screen-time. Model 3, additionally adjusted for loneliness.

*P<0.05; &P<0.01
